# Supplementary material for: To explain or not to explain?—Artificial intelligence explainability in clinical decision support systems
Source: PLOS Digit Health. 2022 Feb 17;1(2):e0000016. doi: 10.1371/journal.pdig.0000016 (PMC9931364; doi:10.1371/journal.pdig.0000016)
Supplement: Attachment — Submitted filename: AuthorResponse_Dec6_JA.docx [file pdig.0000016.s001.docx]

Zurich, December 6th, 2021

**Re:** PDIG-D-21-00082 “To explain or not to explain? - Artificial Intelligence Explainability in Clinical Decision Support”

Dear Professor Horng-Shing Lu,

Thank you very much for the opportunity to revise and resubmit our paper with the modified title «To explain or not to explain? - Artificial Intelligence Explainability in Clinical Decision Support». We greatly appreciate the reviewers' constructive feedback which we believe helped to significantly improve the quality of our work. Please find in the following our point-by-point response to the reviewers’ comments. Changes for each of the points raised have been highlighted in the revised manuscript using track changes.

Thank you very much for receiving our manuscript. We appreciate your time and look forward to hearing from you.

Sincerely,

Julia Amann

**Journal Requirements:**

1. Please provide separate figure files in .tif or .eps format only and remove any figures embedded in your manuscript file. Please ensure that all files are under our size limit of 20MB. Once you've converted your files to .tif or .eps, please also make sure that your figures meet our format requirements For more information about how to convert your figure files please see our guidelines: <https://journals.plos.org/digitalhealth/s/figures>

**Response: Files are provided separately in .tif format.**

2. Please provide a complete Data Availability Statement in the submission form, ensuring you include all necessary access information or a reason for why you are unable to make your data freely accessible. Note that it is not acceptable for the authors to be the sole named individuals responsible for ensuring data access. PLOS defines a study's minimal data set as the underlying data used to reach the conclusions drawn in the manuscript and any additional data required to replicate the reported study findings in their entirety. Any potentially identifying patient information must be fully anonymized. If your research concerns only data provided within your submission, please write "All data are in the manuscript and/or supporting information files" as your Data Availability Statement.

**Response: All data are in the manuscript and/or supporting information files.**

3. We do not publish any copyright or trademark symbols that usually accompany proprietary names, eg (R), (C), or TM (e.g. next to drug or reagent names). Therefore please remove all instances of trademark/copyright symbols throughout the text, including Z-Inspection® on pages 1, 4, 9, 22, and 29.

**Response: We are concerned about this request. While the Z-Inspection process is a pro-bono system without commercial interests, it is a trademarked process to ensure that the quality of the ethical assessment is protected at all times. We used the trademark symbol in accordance with the Guidelines for Trademarks as provided on the** [**journal website**](https://journals.plos.org/digitalhealth/s/licenses-and-copyright#loc-guidelines%20for%20trademarks)**: “Ensure that any reference to a trademark (such as a brand name) is used as an adjective, and not a noun or verb. The trademark should be immediately followed by the generic term for the object that it modifies. Note that because a trademark cannot be used as a noun, it cannot be presented in the possessive or plural form.” We thus resubmit with the trademark symbols still included.**

**Reviewer #1:**

The paper presents an ethical review of arguments for and against explainability for AI-powered CDSS in medicine. The authors present findings from a workshop and draw on a qualitative case study to foster the largely theoretical debate about explanations in medical AI. The subject is topical, clinically and scientifically relevant and overall, the paper is well written.

I enjoyed reading the paper, especially the introduction is well written and well structured. The biggest issue I have with the paper becomes clear from the methods section on, where the paper is confused about the approach, goals and status of the specific case. This is not only methodologically problematic, but also a challenge for assessing the value of the paper and the presented analysis: I do -as it currently stands- not have reasons to trust the claims made in the paper. Below, I outline some specific issues related to this overarching issue.

**Response: We would like to thank the reviewer for their thorough assessment of our work and**

**the constructive feedback provided which we believe helped to significantly strengthen the**

**quality of our paper. Below, please find a point by point response to the reviewer’s comments.**

1. The methodology section lacks information about the followed approach, included data and the analysis of the qualitative data: what information is included in the case study analysis (interviews, observations at case site?) what was provided and discussed at the workshop, who was there, how many participants, how was the data recorded and analyzed, etc.¨

**Response: We agree with the reviewer that the terminology we used to describe our methodological approach may have been misleading and not fully clear. Specifically, we realized that the use of “case study” was misleading. Our paper most closely aligns with the third option proposed by the reviewer (see comment 4). We draw from a use case to develop normative conclusions. In the revised version of the manuscript, we now provide a more detailed description of our approach (see Methodology).**

2. The results do not read as a case study: it is for example not clear when the authors and when the participants are ‘speaking’, whether there was agreement/disagreement and what from that discussion ended up in the paper.

**Response: Thank you for this observation. As pointed out above, we realized that the terminology we used to describe our methodological approach may have led to confusion. In the revised version of the manuscript, we now provide a more detailed description of our methodological approach (see Methodology).**

3. The suggestions in results and discussion are rather general about AI systems and explanation and do not refer to the specific use case, while the description of the specific system is quite specific. How generalizable are suggestions that were discussed for this specific system?

**Response: To advance the currently largely theoretically oriented discourse on explainable AI, we adopted a bottom-up approach where our considerations were driven by a real-world application with the aim to make abstractions for AI-powered CDSS, more broadly. In the results section, there are several references to the use case while in the discussion we focused on abstracting these findings to a more general level. In the revised version of the manuscript, we now make this approach and our motivations clearer (see Methodology).**

4. There are several ways forward: 1) the paper could be rewritten as a more normative paper, then the empirical case should be removed or can be used as an example; 2) The authors clarify the empirical approach and analysis and rewrite the results in such a way that it becomes clear what the participants argued and whether there was (dis)agreement; 3) the authors use the case as a showcase, then the results section should be more specified for this specific case: when and why is explanation needed for this CDSS?

**Response: We greatly appreciate the reviewers’ suggestions and have revised the paper to make our methodological approach clear. Specifically, our paper most closely aligns with the third option proposed by the reviewer. As outlined earlier, we adopted a bottom-up approach to draw normative conclusions from a real-world application (use case) (see Methodology).**

Other issues in the result section:

5. Clarification required: in the paper is described that the AI system has a ‘superior performance’, yet also the high number of false positives is mentioned. Do the authors refer to the sensitivity of the system with superior performance with the superiority? Can the authors clarify whether that an adequate description?

**Response: We agree with the reviewer, the description of “superior performance” is ambiguous. Superior performance in this context was referring to sensitivity and detection time, and we adapted the text to reflect this.**

6. the authors argue that such systems should be able to deal with various accents and dialects, yet do not mention whether that would be a trade-off with for example accuracy? If so, aren’t there alternatives, eg: no recommendation from the system when dialect is not sufficiently understood/training of employees to know the limits of the system?

**Response: We added some information on the challenges for dealing with different dialects and technical possibilities of handling them. Specifically:**

*“If sufficient representation of minorities can not be guaranteed, the system should at*

*least detect when it encounters a not sufficiently understood dialect* [*(66,67)*](https://www.zotero.org/google-docs/?LBe7MJ) *to then*

*notify the dispatchers whilst also refraining from making a prediction.”*

7. authors suggest critical assessment of the systems, for which transparency is suggested to be important. Are there alternatives to this (eg: knowing the reliability of such systems), why is transparancy the best solution?

**Response: We apologize but we are not sure whether we are interpreting the reviewer’s comment correctly. In the scenario outlining the way forward without explainability we precisely explore that there are alternatives which can be considered, i.e. relying on performance alone. Additionally, the discussion outlines several points-of-view on systems without explainability. For example, we argue that when algorithm-driven applications are necessary, explainability might be less relevant than accuracy, in contrast to dedicated decision *support* systems.**

Issues in the discussion and conclusion

8. ‘Taken together, we believe that the current debate around explainability is ‘too theoretical’. It is likely that the question, whether explainability is needed or not and whether explainable or interpretable algorithms are to be preferred, cannot be finally answered on a high and theoretical level.’ This sounds intuitive, but wasn't that the reason to dive into a use-case? What can we learn from this empirical case study that you dived into?

**Response: The quoted consideration is a result of our analysis and points to the need for a bottom-up approach that examines each real-world application *individually* to inform normative needs for explainability in a CDSS. More specifically, the use case we present has drawn our attention to the multiple - and sometimes conflicting - requirements and perspectives present in the clinical setting. This result is also a direct lesson from the examination of the presented use case. This was clarified in the revised manuscript:**

*Rather, we need to individually analyze implemented systems and their specific*

*needs with regards to technology, displayed information, user groups, and the*

*intended use of the system in decision making ADDED: to draw conclusions about*

*explainability requirements. Studying use cases, like the one presented here, can*

*draw attention to the multiple, sometimes conflicting requirements and perspectives*

*present in the clinical setting, which may not emerge from a purely theoretically*

*grounded discourse. (see also comment below)*

9. similar point in the conclusion: ‘ Thus, we deem it likely that the role of explainability cannot be answered definitively at a high, theoretical level.’ Seems an unsatisfying conclusion for an empirically-informed paper.

**Response: We agree with the reviewer that the formulation we chose may have been misleading. We did not want to imply that our analysis did not yield results. On the contrary, it was our intention to highlight our main result: We should not seek a universal answer to the question of whether explainability should be considered a core requirement for AI-powered CDSS or not. We should instead analyze individual real-world applications to uncover areas of concern and to anticipate future ethical and practical challenges and then decide whether this application requires explainability or not. These results also highlight the need for an interdisciplinary approach to studying and assessing AI-powered CDSS given the vast array of stakeholder affected by these solutions and the interdependencies to be considered. This was clarified in the revised manuscript:**

*We deem it likely that the role of explainability cannot be answered definitively at a high, theoretical level. Instead, each system developed for the clinical setting will require an individualized assessment of explainability needs. Thus, this paper cannot provide a universal answer to the question of whether and in what form explainability should be considered a core requirement for AI-powered CDSS. Rather, it illustrates the importance of studying individual real-world applications to uncover areas of concern and to anticipate future ethical and practical challenges. It also highlights the need for an interdisciplinary approach, like the Z-Inspection process, to study and assess AI-powered CDSS given the vast array of stakeholder affected by these solutions and the interdependencies to be considered. This paper presents an example of what such interdisciplinary assessments for explainability requirements could look like in practice.*

10. ‘ Thus, even if overarching plausible arguments exist, such as for explainability as a concept or against black-box explanations, we caution against any absolute dos or don'ts: for example, against legislation that would make explainability a legal requirement or against rules that would impose a ‘ban’ on certain types of algorithms a priori.’ Could you add a more constructive note: what do you suggest using instead?

**Response: A major challenge of the current regulatory pathway is that it was built for old school medical devices that are seldomly updated. We believe that we need a novel regulatory pathway where a potential tool is initially assessed by interdisciplinary advisory boards (incl. patient and public representatives, and representatives of the governing bodies. These boards to assess novel AI-based technologies in very early development and pave the way towards a set of requirements for a specific tool in a specific setting. This is actually a topic that some of the authors of this work currently work on and we would want to make details of this approach public in the respective publication. We have thus amended the manuscript by adding the following paragraph:**

*[...] Instead, we advocate for changes to the regulatory pathways for AI in healthcare medical devices which are currently geared towards tools that are rarely updated. In our view, the current system with one regulatory process for all types of tools will unlikely suffice to guide manufacturers towards the appropriate amount of explainability. It also does not allow for rapid technical amendments in light of retraining and clinical feedback.*

11. ‘ For the given use case, we can thus conclude that the system without well-validated explainability most closely relates to an algorithm-driven tool.’ This doesn't follow from the results as they stand and requires more profound argumentation.

**Response: Thank you for raising this point. In the revised manuscript, we have now further supported our argumentation. Specifically, we argue that without an explanation, dispatchers are, in fact, unable to make an informed decision whether or not to trust and comply with the system’s recommendation. In other words, they only have the choice whether or not to comply and have no information at their disposal to justify this decision. This, in turn, reduces their autonomy. We have added the following to elaborate:**

*This is because without an explanation, dispatchers are, in fact, unable to make an informed decision whether or not to trust and comply with the system’s recommendation. In other words, they only have the choice whether or not to comply and have no information at their disposal to make or justify their decision.*

12. 433‘ The autonomy of the dispatchers to make decisions is greatly reduced.’ This claim is ot supported by the results as they stand

**Response: We agree with the reviewers’ observation that this statement was too strong. In the revised version of the manuscript, we have expanded our argumentation and added the following (see also comment above):**

*This is because without an explanation, dispatchers are, in fact, unable to make an informed decision whether or not to trust and comply with the system’s recommendation. In other words, they only have the choice whether or not to comply and have no information at their disposal to make or justify their decision.*

13. 454 ‘ No matter their level of binding, however, it must be clear what these terms mean. As an example, the current Guidelines for trustworthy AI of the European Union refer to “explicability” as an important concept in making AI transparent (13). It is, however, unclear what this term means, especially as the same document also refers to explainability as a sub-concept.’ Indeed, conceptual clarification is necessary and useful, the following paper presents an attempt to differentiate between transparency and explainability and may be helpful for your analysis: <https://jme.bmj.com/content/47/5/329.info>

**Response: Thank you for pointing us to this paper. While we were not sure whether it fits well in the terminology section, we appreciated the concept of “Computational Reliabilism” framework as a way of fostering trust without explainable AI and referenced it in the section *Explainability: Challenges and Drawbacks* as additional proof to the argument that explanations can be considered less important than performance and validation of the system.**

**Reviewer #2:**

Synopsis of my understanding of the article and its arguments. The paper presents a review of arguments in favor and against explainability for AI-powered Clinical Decision Support Systems (CDSS), employing a qualitative case study and a normative analysis to explore different development scenarios for the CDSS system - with and without explainability. The author's analysis suggests that whether explainability can provide added value to CDSS depends on technical feasibility, the level of validation in case of explainable algorithms, the characteristics of the context in which the system is implemented, the designated role in the decision-making process, and the key user group(s). The authors conclude that each CDSS will require an individualized assessment of explainability needs.

Is the submission original and provocative? Is its argument made in a coherent and succinct manner? Is it suitable for PLOS Digital Health? The submission is somewhat provocative, arguments are clear, and over-all this article fits the theme of PLOS DigitalHealth. The article is building upon the theme of trying to explain algorithms in various domains, including healthcare. The reviewer believes this paper addresses an inherently interesting topic: a real-world account of the role of explainability (here, an AI system used to detect cardiac arrest in an emergency call setting in Denmark). The following comments are suggestions for making improvements on an already solid paper.

1. What are the strengths and weaknesses of the submission?
   Strengths: Thank you for addressing the issue of defining explainability, especially the nuance between explainable and interpretable algorithms.
   Thank you for discussing the technical challenges inherent in explainability, including the all-important human (here, the HCP, dispatcher) requirement of trust.

Weaknesses: My main criticism of the article relates to the assertion in the title: "To explain or not to explain? . . ." Are you arguing that explanations may not be necessary? Or are you arguing that explanations are necessary, but time, user, and context specific. I interpret your paper as pursuing the latter. For example, is explainability, in your assessment, real-time explainability (like in an emergency situation) or can explainability be time-shifted (like during a training)?

**Response: In our opinion, the title does not include any assertion. On the contrary, we deliberately did not choose a title that would give away any results of the paper but formulated the title as an open question to raise interest in our paper. We believe that is a valid and scientifically common approach. Our results and arguments then become evident once the reader explores the abstract or reads the full paper.**

2. Finally, I am having trouble with the last sentence. “Lastly, our paper demonstrates that interdisciplinary assessments like the Z-Inspection® process are invaluable tools to analyse the characteristics and the impact of AI-based CDSSs.” I tend to agree with this, but I’m not sure that your paper is demonstrating this process as “invaluable.”

**Response: We agree with the reviewer’s comment and have rephrased this section:**

*Thus, this paper cannot provide a universal answer to the question of whether and in what form explainability should be considered a core requirement for AI-powered CDSS or not. Rather, it illustrates the importance of studying individual real-world applications to uncover areas of concern and to anticipate future ethical and practical challenges. It also highlights the need for an interdisciplinary approach, like the Z-Inspection process, to studying and assessing AI-powered CDSS given the vast array of stakeholder affected by these solutions and the interdependencies to be considered. This paper presents an example of what such interdisciplinary assessments for explainability requirements could look like in practice.*

Could improvements and revisions (minor, major) address the weaknesses identified? If so, please provide a specific listing of such improvements to support such a recommendation. See above.

3. Reviewer’s Boilerplate Comments

The title of the article is not totally clear, and the abstract could be more informative.

**Response:** **We slightly adapted the title and abstract in line with the reviewers’ suggestions.**

*Title: To explain or not to explain? - Artificial Intelligence Explainability in Clinical Decision Support Systems*
*Explainability for artificial intelligence (AI) in medicine is a hotly debated topic. Our paper presents a review of the key arguments in favor and against explainability for AI-powered Clinical Decision Support System (CDSS) applied to a concrete use case, namely an AI-powered CDSS currently used in the emergency call setting to identify patients with life-threatening cardiac arrest. More specifically, we performed a normative analysis using socio-technical scenarios to provide a nuanced account of the role of explainability for CDSSs for the concrete use case, allowing for abstractions to a more general level. Our analysis focused on three layers: technical considerations, human factors, and the designated system role in decision-making. Our findings suggest that whether explainability can provide added value to CDSS depends on several key questions: technical feasibility, the level of validation in case of explainable algorithms, the characteristics of the context in which the system is implemented, the designated role in the decision-making process, and the key user group(s). Thus, each CDSS will require an individualized assessment of explainability needs and we provide an example of how such an assessment could look like in practice.*

The text is generally well-written, free of unnecessary jargon, and streamlined.

The significance of the problem in the paper is explained and the significance is compelling.

The ideas are developed logically and thoroughly.

Different viewpoints could be better acknowledged.

**Response:** **We added additional references (highlighted in text), some of which were suggested by reviewer 1 to better acknowledge different viewpoints.**

Sources are well-integrated into the paper (not for the sake of adding sources).

Word choice could be more specific and concrete (see comment on "To explain or not to explain?)

**Response:** **We realized that the word choice, particularly in the methodology section may have been misleading, as also pointed out by reviewer 1. In the revised version of the manuscript, we aimed for a more concrete word choice to improve clarity.**

Sentences are clear.

Overall organization of the argument is effective.

No grammatical errors.

**Reviewer #3:**

The authors seek to find out the role explainability plays in the adaptation/performance of clinical decision support systems. The results are based on a Z-inspection process where some of the authors in this paper developed, and four consecutive workshops assessing the truthworthiness of AI systems, using an AI-based CDSS system to recognize out-of-hospital cardiac arrest from audio files of calls to the emergency medical dispatch center (reference 31). The authors took the outcomes of the workshops and analyzed them focusing on the issue of explainability. The reviewer note that distinguishing the case of employing interpretable algorithms from the case of block-box algorithms amended with an explanation algorithms is very important point. Also the three layers approach to deliberate and present the findings is quite fruitful. The results in this paper are helpful for people working/debating the explainability of AI machine algorithms, instructive for people working on assessing AI algorithms, however, for people who want to understand the process of deriving the results from inputs which is the outcome of reference 31, they do encounter some obstacles.

**We would like to thank the reviewer for their positive evaluation of our work and the suggestions for improvement. However, we would like to clarify a point regarding our methodological approach. Based on the reviewers’ comments, we came to realize that the terminology we used to describe our methodological approach, specifically the term “case study”, may have been misleading and not fully clear. Our analysis is, in fact, based on the initial assessment of the system and the consequent mapping of ethical issues and tensions, rather than the workshops themselves (which served as the basis for the initial assessment). This also influences our answer to the second point that the reviewer raised (see below).**

The reviewer would like to suggest:

1. The authors briefly recap reference 31 for readers to get a better pictures.

**Response:** **We agree with the reviewer's suggestion. In line also with comments by reviewer 1, the revised manuscript now presents a more detailed description of our methodological approach, including additional information from reference 31, as suggested by the reviewer (see Methodology).**

2. Give some quotations from the workshops to support summarized arguments/observations.

**Response:** **We believe that this suggestion was motivated by the lack of clarity in our initial methodological description. The resubmitted version clarifies that our analysis is based on the initial z-inspection assessment of the system and the consequent mapping of ethical issues and not the workshops themselves. In line with suggestions by reviewer 1, we revised the methods section and clarified how the initial assessment of the AI system informed our analysis. Specifically, the initial assessment of the system and consequent mapping of ethical issues led to identification of lack of explainability as an ethical issue and possible explanation for the lack of dispatchers' compliance with the system’s recommendations. This constituted the point of departure for our analysis.
We believe that identifying quotes from the workshops post hoc would include a considerable bias that would not do our analysis justice.
In the revised manuscript, the workshop outcomes - the ethical issues and tensions - are summarized now in figure 3. For a detailed description of the assessment and how the ethical issues were conceived we refer, here and in the paper, to the corresponding publication** *Zicari et al., “On Assessing Trustworthy AI in Healthcare. Machine Learning as a Supportive Tool to Recognize Cardiac Arrest in Emergency Calls”. Front. Hum. Dyn., Human and Artificial Collaboration for Medical Best Practices, 08 July 2021*
